# Correlational Analysis of the Physicochemical Indexes, Volatile Flavor Components, and Microbial Communities of High-Temperature *Daqu* in the Northern Region of China

**DOI:** 10.3390/foods12020326

**Published:** 2023-01-09

**Authors:** Zemin Pang, Weiwei Li, Jing Hao, Youqiang Xu, Binghao Du, Chengnan Zhang, Kun Wang, Hua Zhu, Hongan Wang, Xiuting Li, Changhong Guo

**Affiliations:** 1 Key Laboratory of Molecular and Cytogenetic, College of Life Science and Technology, Harbin Normal University, Harbin 150025, China; 2 Key Laboratory of Brewing Microbiome and Enzymatic Molecular Engineering, China General Chamber of Commerce, Beijing Technology and Business University, Beijing 100048, China; 3 Beijing Huadu Wine Food Limited Liability Company, Beijing 102212, China

**Keywords:** high-temperature *Daqu*, physicochemical indexes, volatile flavor components, microbial community

## Abstract

*Daqu* is a microbial-rich baijiu fermentation starter. The high-temperature *Daqu* plays an essential role in the manufacturing of sauce-flavored baijiu. However, few studies have focused on three kinds of high-temperature *Daqu* (white, yellow, and black *Daqu*) in northern China. In this study, the physicochemical indexes, volatile flavor compounds, and microbial characteristics of the three different colors of high-temperature *Daqu* in northern China were comparatively analyzed to reveal their potential functions. White *Daqu* (WQ) exhibited the highest liquefying power and starch, and black *Daqu* (BQ) showed the highest saccharifying and esterifying powers. A total of 96 volatile components were identified in the three types of *Daqu*, and the contents of the volatile components of yellow *Daqu* (YQ) were the highest. The microbial community structure analysis showed that *Bacillus* and *Byssochlamys* were dominant in BQ, *Kroppenstedtia* and *Thermoascus* were dominant in WQ, and *Virgibacillus* and *Thermomyces* dominated the YQ. The RDA analysis revealed the correlation between the dominant microorganisms and different physicochemical indexes. The Spearman correlation analysis indicated that *Oceanobacillus*, *Saccharopolyspora*, *Staphylococcus*, *Pseudogracilibacillus*, *Byssochlamys,* and *Thermomyces* showed positive correlations with part of the majority of the key volatile flavor compounds. This work provides a scientific basis for the actual production of different colors of high-temperature *Daqu* in the northern region of China for sauce-flavored baijiu.

## 1. Introduction

Chinese baijiu is one of the oldest distilled spirits in the world, with a history of a thousand years and is an indispensable part of Chinese culture [1]. Sorghum and other grains are used as the raw materials in the production of baijiu, which is then processed using a combination of solid-state fermentation and steaming distillation methods to generate diverse types of baijiu [2]. According to its unique brewing technology and distinctive flavor characteristics, baijiu is classified into twelve flavor types [1]. The three primary flavor categories of baijiu are considered to be sauce-, strong-, and light-flavored baijiu [3,4]. Among the three categories, the sauce-flavored baijiu brewing technique has the most intricate and distinctive manufacturing stages; it consists primarily of producing *Daqu*, then solid-state fermentation, distilling, storing and aging, and blending [5]. Therefore, sauce-flavored baijiu is known for its strong soy sauce flavor, full-bodied, long aftertaste, and long-lasting fragrance, which is widely loved by consumers [6]. *Daqu* is a baijiu fermentation starter affecting solid-state fermentation and the quality and flavor of baijiu, which has multi-microorganisms [4]. In addition to metabolically synthesizing a variety of flavor compounds, the microbes in *Daqu* also break down some of the hazardous chemicals present in the raw materials. [7,8,9]. Therefore, *Daqu* plays an essential role in the manufacturing of sauce-flavored baijiu [10,11].

**Figure 1:**
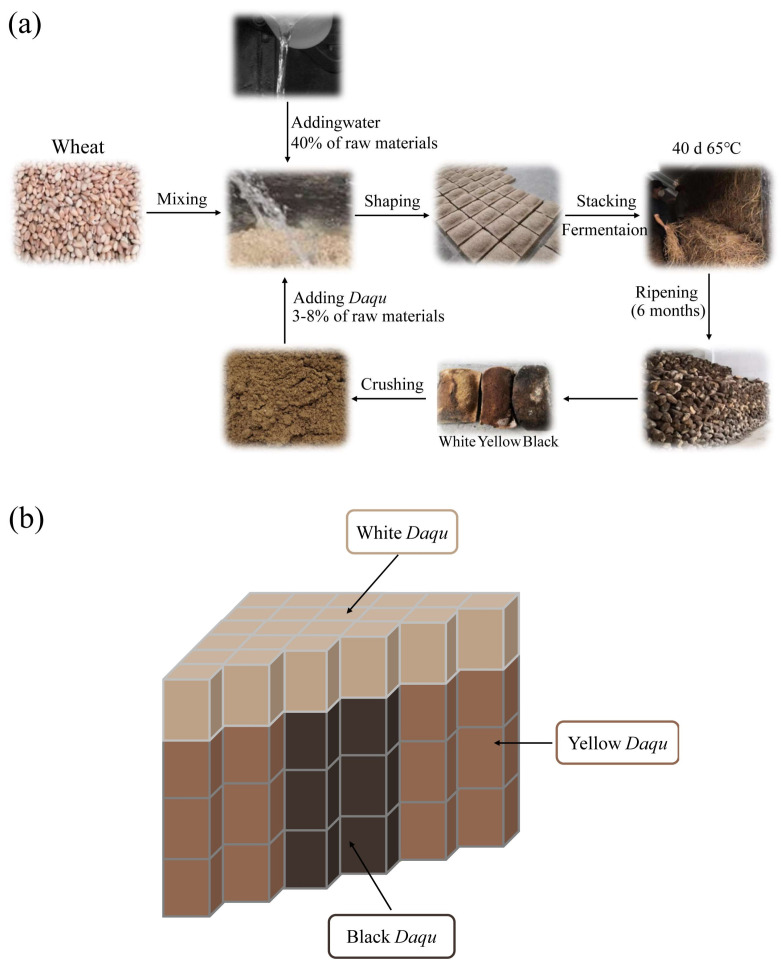
Preparation of *Daqu* for sauce-flavored baijiu. (**a**) The manufacturing process of *Daqu* for sauce-flavored baijiu. (**b**) Characteristic diagram of the three different types of high-temperature *Daqu*.

Depending on the different temperatures of the *Daqu*, it could be divided into three categories: low-temperature (45–50 °C), middle-temperature (50–60 °C), and high-temperature *Daqu* (60–65 °C) [12]. Different types of *Daqu* are suitable for the fermentation of baijiu with various flavors. High-temperature *Daqu* serves as the fermenting starter of sauce-flavored baijiu, which is created through the intricate solid-state fermentation of wheat (Figure 1a), which involves moistening wheat, followed by mixing, shaping, stacking and fermentation, and ripening [2]. *Daqu* fermentation is usually undertaken in an open environment, with microorganisms from the wheat, air, ground, water, production tools, and operators colonized in it [13]. Furthermore, the condition of high-temperature fermentation is conducive to forming a unique microbial system in *Daqu* and thus provides a wide variety of enzymes and volatile compounds, which has key roles in enriching the flavor of sauce-flavored baijiu [14,15]. During the *Daqu* manufacturing process, due to the different stacking positions of *Daqu* lumps and environmental factors, such as the temperature in the Qu-room, which are usually divided into three kinds, i.e., white, yellow and black *Daqu*, according to their color divergence [16,17]. The top layer of *Daqu* samples (white *Daqu*) is at a relatively low temperature (accounting for ~10%), the *Daqu* lumps in the middle layer (yellow *Daqu*) are at a moderate temperature (accounting for ~80%), and the core layer *Daqu* lumps (black *Daqu*) are at the high temperature (accounting for ~10%) [17] (the simulation diagram is shown in Figure 1b). However, there is no fixed ratio for the use of the three colors of *Daqu* in the fermentation process of sauce-flavored baijiu, which is primarily based on the working experience of the workers.

Sauce-flavored baijiu manufacturing areas mainly include the Guizhou, Sichuan, Shandong, and Heilongjiang provinces, as well as Beijing and Tianjin city, and among them, the Guizhou province is the representative manufacturing region [18]. Studies have indicated that the flavor and taste of sauce-flavored baijiu produced in Beijing, Shandong, and other northern production areas are distinctive when compared with Sichuan, Guizhou, and other southern production areas [19]. Some of the main reasons are that the added *Daqu* is different, the microbial composition in *Daqu* is usually distinguished between different producing regions, and this generates different microbial ecologies during the fermentation process and different microbial flavor metabolites of the products [4,20]. Meanwhile, the microbial metabolites during the *Daqu* manufacturing process are also a significant route for the flavor chemical composition of the final baijiu products [21]. Consequently, it is quite important to elucidate the microbiome community composition and potential functions of northern high-temperature *Daqu* to resolve the flavor differences between southern and northern sauce-flavored baijiu.

At present, researchers have explored the microbiota and different metabolites in different types of *Daqu*, such as Chinese te-flavor baijiu *Daqu*, high-temperature *Daqu,* low-temperature *Daqu*, and Nongxiangxing *Daqu,* using a combination of microbiology and physicochemical properties [22,23,24,25]. However, the available studies on the samples of high-temperature *Daqu* are mainly from southern producing areas, such as Guizhou and Sichuan. For example, recently, Cai et al. used high-throughput sequencing and electronic senses (e-senses) to elucidate the relationship between the fungal communities and flavor features of different types of high-temperature *Daqu* from the Hubei province [26]. Shi et al. utilized high-throughput sequencing technology combined with co-occurrence network analysis and PICRUSt to illustrate the potential functions of different colors of high-temperature *Daqu* in the Guizhou province [27]. The Chinese brewing industry is spread over the north and south of the country, and there are some very distinctive sauce-flavored baijiu production enterprises in the north, but the functions of three colored high-temperature *Daqu* are still unknown to the workers in actual production. Therefore, it is an urgent need to clarify the microbial community diversity and physicochemical properties of the three colors of high-temperature *Daqu* from the northern regions of China.

In the current work, we mainly focused on the characteristics of high-temperature *Daqu* with different colors in Beijing. The physicochemical properties and microbial community diversities of the three kinds of *Daqu* were compared and analyzed through a combination of high-throughput sequencing technology, and the volatile flavor components were detected using headspace solid-phase microextraction gas-chromatographic mass- spectrometer (HS-SPME-GC-MS). The correlation between the dominant microorganisms and physicochemical indexes and the volatile compounds revealed the function of *Daqu* microorganisms.

## 2. Materials and Methods

### 2.1. Sampling

The three colors of high-temperature *Daqu* were randomly collected in triplicates from Beijing Huadu Brewery and Food Industry Co., Ltd., Beijing, China, marked as white *Daqu* (WQ), black *Daqu* (BQ), and yellow *Daqu* (YQ). The subject of our study was matured *Daqu,* which was stored for six months after fermentation. Each type of *Daqu* was completely crushed into fine powders as the experimental samples. Moreover, a portion of the samples was stored at 4 °C for the analysis of the physicochemical indexes and volatile flavor components, while the remaining portion of the samples was stored at −80 °C until the genomic DNA extraction before PCR amplification.

### 2.2. Analysis of the Physicochemical Indexes

The physicochemical indexes of three *Daqu* samples were determined according to the general analytical method BQ/T 4257-2011 of brewing *Daqu* in the light industry of the People’s Republic of China [28]. These physicochemical indexes included moisture, acidity, starch, saccharifying power, liquefying power, esterifying power, and fermenting power.

### 2.3. Analyzing the Volatile Compounds

The flavor compounds were detected with an SPME fiber (50/30 μm divinylbenzene/carboxen/polydimethylsiloxane, DVB/CAR/PDMS, Supelco, Inc., Bellefonte, PA, USA) [29]. The procedure was as follows: 1 g of three *Daqu* samples, 5 mL of saturated NaCl solution, and 4-octanol (a final concentration of 5 mg/L) were packaged into a 15 mL headspace bottle and covered with a lid. Subsequently, the bottle was placed in a water bath at 50 °C for 10 min, and the fiber head of the SPME was inserted into the bottle for 30 min at 50 °C. Following the extraction, the fiber was placed into the gas chromatography-mass spectrometer (GC-MS) (Trace MS/GC, Thermo Quest Finnigan Co., Silicon Valley, CA, USA) injector for thermal desorption for 5 min, and the desorbed volatile compounds were examined using GC-MS.

The following operating parameters were used for the GC: a split ratio of 20:1 and an inlet temperature of 250 °C. High-purity helium served as the carrier gas, flowing at a rate of 1 mL/min through a DB-WAX column (30 m × 0.25 mm × 0.25 μm, Agilent Technology, CA, United States). The oven temperature was held at 40 °C for 5 min, raised to 100 °C at a rate of 5 °C/min, and held for 10 min, raised to 150 °C at a rate of 10 °C for the last 10 min, increased to 250 °C at a rate of 10 °C for the last 10 min. The MS condition was as follows: The temperature of the interface was set at 250°C, the electron impact mode (EI) was at 70 eV, the ion sources were at 230°C, and the MS scanning was from 30 to 450 amu.

### 2.4. Extraction of the Genomic DNA, PCR Amplification, and Illumina MiSeq Sequencing

The *Daqu* samples’ genomic DNA was extracted using a PowerSoil DNA Isolation Kit (Mo-Bio, Carlsbad, CA, USA) according to the manufacturer’s instructions, and then electrophoresis was performed on a 1% agarose gel, which checked the DNA quality. The bacteria were identified via PCR amplification of the v3-v4 region of the 16S rRNA gene, using the primers 338F (5′-ACTCCTACGGGAGGCAGCAG-3′) and 806R (5′-GGACTACHVGGGTWTCTAAT-3′) [30] and the internal transcribed spacer (ITS1) regions of the fungal rRNA genes were amplified using the primers ITS1F (5′-CTTGGTCATTTAGAGGAAGTAA-3′) and ITS1R (5′-GCTGCGTTCTTCATCGAT GC-3′) [30]. The PCR products were sequenced on an Illumina MiSeq PE300 platform/NovaSeq PE250 platform (Illumina, San Diego, CA, USA), according to the standard protocols by Majorbio Bio-Pharm Technology Co., Ltd. (Shanghai, China).

### 2.5. Data Analysis

The raw gene sequencing reads were demultiplexed, quality-filtered using Trimmomatic [31], and merged using FLASH software (version 1.2.11, Johns Hopkins University, Baltimore, MD, USA) [32]. The operational taxonomic units (OTUs) with a 97% similarity cutoff were clustered using UPARSE (version 7.1, Taxon Biosciences, Inc., Tiburon, CA, USA) [33]. Moreover, the UCHIME algorithm was used to determine and eliminate the chimeric sequences. The OTU-based assessments of the alpha diversity indexes, including community richness (Chao1, ACE) and community diversity (Shannon, Simpson) and coverage, were conducted by Mothur [34]. The distribution between the samples and microbial communities was predicted and visualized using a co-occurrence network. A redundancy analysis (RDA) was carried out to reveal the correlations between the microbial genera and physicochemical indexes by using Canoco 5 software (Chinese Academy of Sciences, Bejing, China) [35]. The Spearman correlations between the volatile compounds and microbiological genera were shown on the correlation heat maps drawn with OriginPro 2022 (OriginLab Corporation, MA, USA) and heatmap package software (version 1.0.12, University of Alberta, Edmonton, AB, Canada.). All statistical analyses were performed with SPSS26.0 (SPSS Inc., Chicago, IL, USA).

## 3. Results

### 3.1. Analysis of the Physicochemical Indexes

The physicochemical indexes of *Daqu* determine its quality and whether it is suitable for fermentation, and these parameters also influence microbial functions in *Daqu* [17]. Table 1 demonstrates the discrepancies in the physicochemical indexes of three samples. YQ exhibited the lowest esterifying power (198.37 ± 17.50 U), and the other physicochemical parameters were at a medium level. WQ had the highest liquefying power (0.18 ± 0.01 U) and starch (64.71%), whereas the moisture (10.99%) was the lowest. BQ showed the highest moisture (13.28%), saccharifying power (180.80 ± 7.16 U), and esterifying power (288.36 ± 14.61 U), and the lowest starch (59.26%). These samples were not significantly different in terms of acidity and fermenting power. These results revealed the differences in the physicochemical indexes in three types of *Daqu*.

### 3.2. Analysis of the Volatile Flavor Compounds in the Three Daqu Samples

**Figure 2:**
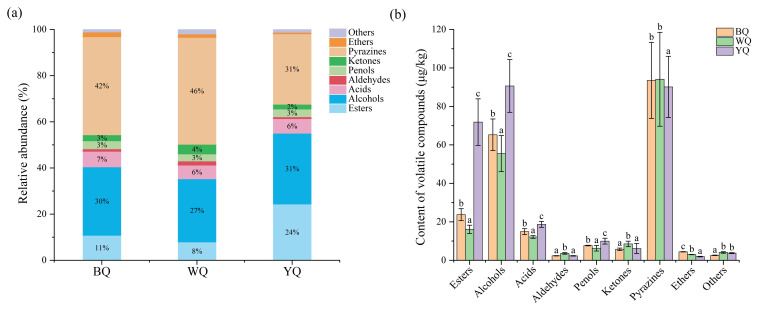
Different kinds of volatile compounds in three *Daqu* types. (**a**) Proportion of different volatile compounds. (**b**) Contents of various volatile compounds. The different letters represent the significant differences in the contents of volatile compounds between the samples (*p* < 0.05).

The quality and aroma characteristics of *Daqu*, particularly in terms of flavor, are closely tied to their quality in sauce-flavored baijiu. A total of 96 volatile compounds were detected in three samples; BQ contained 55, WQ contained 59, and YQ contained 61. As indicated in Table S1, they were categorized into seven groups based on their chemical structural properties, including esters (19), alcohols (16), acids (16), aldehydes (5), phenols (7), ketones (5), pyrazines (7) and ethers (4), as well as others (17). Similarly to *Daqu* for manufacturing strong- and light-flavored baijiu [36,37], esters were also dominant volatile compounds in high-temperature *Daqu*. Moreover, alcohols, acids, pyrazines, and phenols were also the dominant volatile compounds (Figure 2a). Figure 2b illustrates the concentrations of distinct volatile components in several samples. The highest ester content in YQ (71.86 ± 12.11 μg/kg) was 4.46 times higher than the lowest content in WQ (16.11 ± 2.15 μg/kg), and the ethyl acetate contents in YQ (10.65 ± 3.32 μg/kg) and BQ (8.76 ± 2.44 μg/kg) were significantly higher than in WQ (1.14 ± 0.02 μg/kg). In addition, the content of phenethyl acetate (12.77 ± 2.24 μg/kg), hexadecanoic acid ethyl ester (19.78 ± 4.53 μg/kg), and n-propyl 9,12-octadecadienoate (13.03 ± 0.71 μg/kg) also were the highest in YQ. The alcohol content was significantly higher in YQ (90.66 ± 13.73 μg/kg) and BQ (65.25 ± 8.23 μg/kg) than in WQ (55.49 ± 9.40 μg/kg). Amongst the alcohols, the phenethyl alcohol content in YQ (57.64 ± 8.31 μg/kg) was the highest, and the 2,3-butanediol content in WQ (17.13 ± 4.18 μg/kg) was the highest. The pyrazines were predominant volatile compounds in the three types of *Daqu*, whose contents were 93.56 ± 19.77, 94.12 ± 24.41, and 90.15 ± 15.89 μg/kg in BQ, WQ, and YQ, respectively. Amongst the pyrazines, tetramethylpyrazine accounted for 31-46% of the total pyrazines, which was the most abundant. The contents of different types of volatile compounds showed significant discrepancies in the different types of *Daqu*. It is worth noting that the volatile compound content in YQ was the highest among the three samples. 

### 3.3. Analysis of the Microbial Community Composition and Diversity

#### 3.3.1. Microbial Diversity Analysis in the Three *Daqu* Samples

The bacterial 16S rDNA fragment effective sequences of the three samples were in the range of 70,389–108,510, and the fungi ITS DNA fragment effective sequences were in the range of 74,457–123,470; these sequences in the samples were classified into OTUs with 97% similarity. The diversity of different samples was analyzed after normalization. Table 2 lists the α-diversity indexes of the microbial communities, including the species richness (Chao1 and ACE), diversity (Shannon and Simpson), and coverage index. All samples had coverage of 99.9%, which indicated that the samples were reasonable. The species richness and diversity of the bacterial levels in the BQ and YQ samples exhibited similar trends, which were higher than in the WQ sample. At the fungi level, there was also no significant difference in species richness and diversity between the BQ and YQ samples. It suggested that compared with the WQ sample, BQ and YQ exhibited greater microbial species richness and diversity. Moreover, the bacteria in the samples had more species richness and variety than fungi.

#### 3.3.2. Analysis of the Microbial Community Composition and Diversity

**Figure 3:**
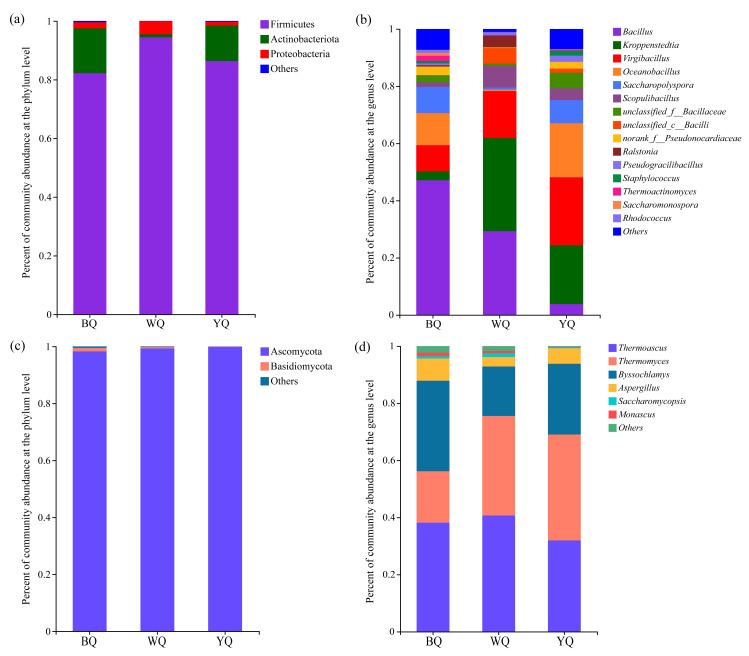
Microbial community composition of each sample. (**a**) Diagram for the bacteria according to the phylum level. (**b**) Diagram for the bacteria according to the genus level. (**c**) Diagram for the fungi according to the phylum level. (**d**) Diagram for the fungi according to the genus level.

To clarify the compositions of the microbial communities in three types of high-temperature *Daqu*, the phylum, genus, and species level were analyzed, and an average relative abundance of over 1.00% was defined as the dominant phyla, genera, or species. The dissimilar microbial community profiles are shown in Figure 3. At the bacterial phylum level, Firmicutes, Actinobacteria, and Proteobacteria were prevalent bacterial phyla. (Figure 3a). Overall, Firmicutes was the most abundant in the WQ sample (94.42%), BQ sample (82.21%) and YQ sample (86.33%), and Actinobacteria was significantly higher in the BQ sample (15.29%) than in the YQ sample (12.10%), and the WQ sample (1.07%) was the lowest. Proteobacteria were in the BQ (2.06%), WQ (4.46%), and YQ (1.31%) samples. At the bacterial genus level (Figure 3b), although the bacteria in the three samples were different, *Bacillus*, *Kroppenstedtia*, *Virgibacillus*, *Oceanobacillus*, and *Scopulibacillus* were prominent bacteria in the three *Daqu* samples. The dominant bacteria in the BQ sample included *Bacillus* (47.11%), *Oceanobacillus* (11.20%), *Virgibacillus* (9.17%), *Kroppenstedtia* (3.08%), *Thermoactinomyces* (1.84%), *norank_f_Pseudonocardiaceae* (3.00%), *unclassified_C_Bacillaceae* (2.55%), *Scopulibacillus* (1.54%), *Rhodococcus* (1.10%), and *Saccharopolyspora* (1.01%). Sample WQ’s dominant bacteria were *Kroppenstedtia* (32.65%), *Bacillus* (29.29%), *Virgibacillus* (16.36%), *Scopulibacillus* (7.58%), *unclassified _C_ Baclli* (5.45%), *Pseudogracilibacillus* (1.08%) and *Ralstonia* (4.20%). Primarily, the bacteria in the YQ sample included *Virgibacillus* (23.80%), *Kroppenstedtia* (20.55%), *Oceanobacillus* (18.88%), *Saccharopolyspora* (8.10%), *unclassified_f_Bacillaceae* (5.46%), *Scopulibacillus* (4.16%), *Bacillus* (3.79%), *norank_f_Pseudonocardiaceae* (2.38%), *Pseudogracilibacillus* (2.11%), *Staphylococcus* (1.71%), and *unclassified_C_Bacilli* (1.42%). Figure S1a shows the results at the species level; *Bacillus_smithii* within the genus *Bacillus* accounted for 34.78% and 27.20% bacterial abundance in the BQ and YQ samples, much higher than WQ (0.06%). These results revealed that the compositions of the bacterial communities in different *Daqu* types were significantly different. 

At the fungal phylum level, Ascomycota was the predominant fungal phylum, and the Ascomycetes level was nearly 100% in the BQ (98.26%), WQ (99.29%), and YQ (99.85%) samples (Figure 3c). Figure 3d displays the differences in the three samples at the level of the fungal genus. The genera of *Thermoascus*, *Thermomyces*, *Byssochlamys,* and *Aspergillus* were dominant in all samples. The abundances of *Thermoascus* were 38.15%, 40.68%, and 31.95% in the BQ, WQ, and YQ samples, respectively. *Thermomyces* was also present in the BQ sample (18.03%) and in WQ (34.86%) and YQ (37.10%). The abundances of *Byssochlamys* were 31.74%, 17.31%, and 24.78% in BQ, WQ, and YQ, respectively. *Aspergillus* in BQ, YQ, and WQ accounted for 7.77%, 3.29%, and 5.45%, and *Saccharomycopsis* in BQ, YQ, and WQ accounted for 0.84%, 1.32%, and 0.30%, while *Monascus* in BQ, YQ, and WQ, accounted for 1.22%, 0.85%, and 0.15%. It should be emphasized that high-temperature types of *Daqu* were also found to include *Saccharomycopsis* and *Monascus*, both of which had low relative abundances (1%) but were essential for the fermentation of baijiu. At the fungal species level, *Thermomyces_lanuginosus* within the genus *Thermomyces* accounted for 37.09% and 34.83% fungal abundances in YQ and WQ. *Thermoascus_crustaceus* within the genus *Thermoascus* had the highest proportion in YQ (31.86%). Lastly, *Byssochlamys_spectabilis* within the genus *Byssochlamys* occupied the highest proportion of the BQ sample (31.71%) (Figure S1b).

#### 3.3.3. Analysis of the Common and Unique Microorganisms in the Three *Daqu* Samples

**Figure 4:**
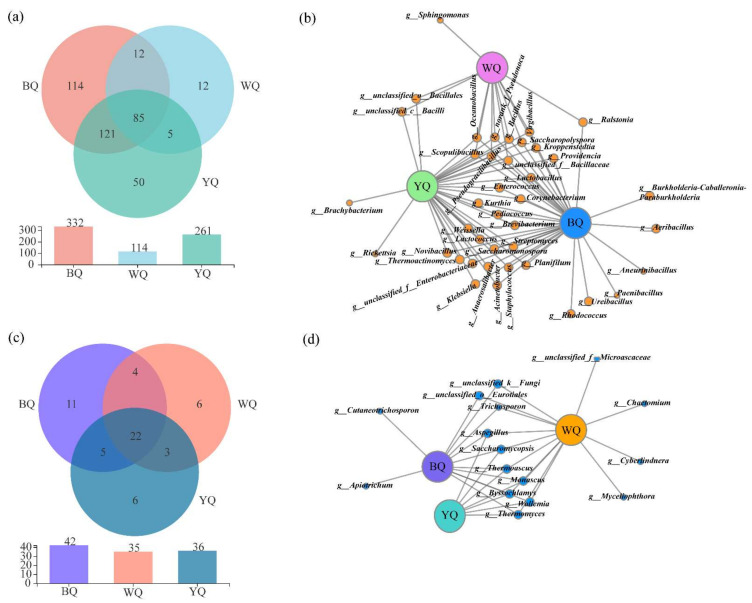
Venn diagram of the OTUs; (**a**) bacteria and (**c**) fungi. The network graphs show the common and unique (**b**) bacterial and (**d**) fungal genera.

A Venn diagram of Figure 4a,c was created based on the samples, which was used to determine whether solely shared OTUs existed. The three *Daqu* samples had a total of 399 bacterial OTUs; sample BQ had 332, sample WQ had 114, and sample YQ had 261 bacterial OTUs, respectively. Meanwhile, the three *Daqu* samples included a total of 57 fungal OTUs; sample BQ had 42, sample WQ contained 35, and sample YQ contained 36. In terms of bacteria and fungi, sample BQ included the most OTUs, while sample WQ contained the fewest. There were 85 common bacterial OTUs and 22 common fungal OTUs in the three samples, and each sample had its own unique bacterial and fungal OTUs. The independent bacterial OTUs in the BQ sample (114) were the highest and far exceeded the YQ (50) and WQ (12) samples, while the OTUs of the independent fungi were less in the three samples. Thus, it could be seen that the bacterial communities of the three *Daqu* samples may have included a range of distinct bacterial species with relatively high abundances, while the fungal communities may have had a range of distinct fungal species with relatively low abundances.

A co-occurrence network analysis could be used to show the distribution between the samples and species. It is possible to determine the co-existence connections of species in the environmental samples using a correlational analysis of the species abundance information between various samples, which may be used to highlight the similarities and differences between the samples. (Figure 4b,d). In the network, the edge connecting two nodes represents the pairwise relationship between them. The samples with species abundances in the top 50 were selected for analysis. At the bacterial genus level, three types of samples had nine common genera; BQ and YQ had twenty-eight common genera, BQ and WQ had one common genus, and YQ and WQ had two common genera. Furthermore, each sample had its own unique genera; BQ had six species, YQ had two genera, and WQ only had one genus. At the fungal genus level, three types of samples had seven common genera; BQ and WQ had three common genera, and there were two unique genera in BQ and four in WQ. The results proved once again that the bacterial communities of the BQ YQ samples are more similar.

### 3.4. Correlation between the Physicochemical Indexes and Microbial Communities of the Daqu Samples

**Figure 5:**
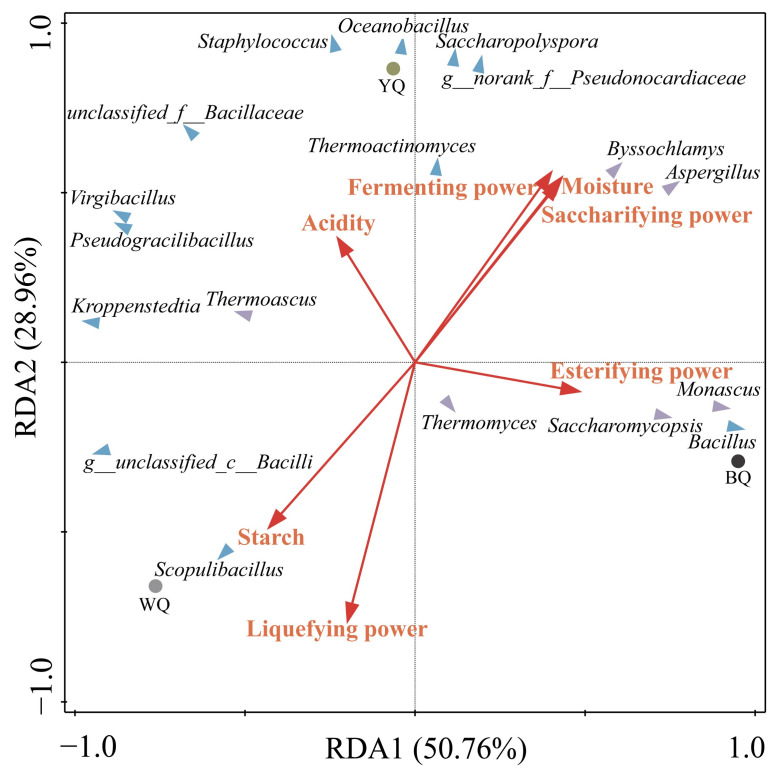
Redundancy analysis (RDA) of the different genera and physicochemical indexes.

The potential correlations between twelve bacterial and six fungal genera and seven physicochemical indexes were mapped using an RDA analysis of the three types of *Daqu*. As shown in Figure 5, the liquefying power positively correlated with *Thermomyces*. The esterifying power positively correlated with *Bacillus*, *Saccharomycopsis*, and *Monascus* near BQ. The saccharifying and fermenting powers showed significantly positive correlations with *Aspergillus* and *Byssochlamys*. Starch exhibited a significantly positive correlation with *Scopulibacillus* near WQ. The acidity was strongly positively correlated with *unclassified_f__Bacillaceae*.

### 3.5. Correlation between the Volatile Flavor Components and Microbial Communities of Daqu Samples

**Figure 6:**
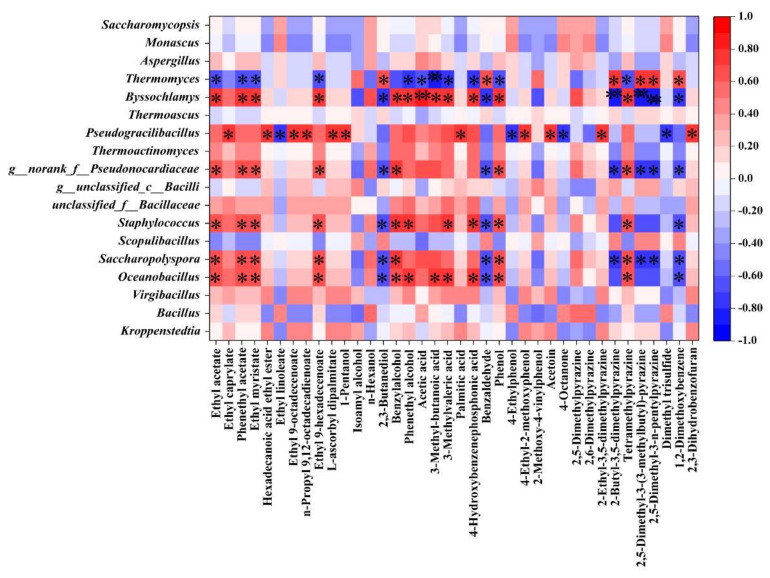
Correlation heatmap between the key flavor compounds and dominant genera. The asterisks show significant correlations (different flavor compounds in Table S1; “*”, 0.01 < *p* < 0.05; “**”, 0.001 < *p* < 0.01; “***”, *p* < 0.001).

Spearman’s correlation analysis was used to evaluate the potentially associated relationship between the key volatile compounds and microorganisms (microbial genera) in different *Daqu* samples. The results in Figure 6 revealed that the bacterial genera *Oceanobacillus*, *Saccharopolyspora*, and *g__norank_f__Pseudonocardiaceae Staphylococcus* and fungi genus *Byssochlamys* were positively correlated with four esters (ethyl acetate, phenethyl acetate, hexadecanoic acid ethyl ester, and ethyl 9-hexadecenoate), two alcohols (benzylalcohol and phenethyl alcohol), four acids (acetic acid, 3-methyl-butanoic acid, 3-methylvaleric acid, and 4-ydroxybenzenephosphonic acid), phenol, and tetramethylpyrazine. However, *Thermomyces* was negatively correlated with these compounds, and it was positively correlated with 2,3-butanediol, benzaldehyde, three pyrazines (2-butyl-3,5-dimethylpyrazine, 2,5-dimethyl-3-(3-methylbutyl)-pyrazine, and 2,5-dimethyl-3-n-pentylpyrazine) and 1,2-dimethoxybenzene. *Pseudogracilibacillus* was closely related to five esters (ethyl caprylate, hexadecanoic acid ethyl ester, ethyl 9-octadecenoate, n-propyl 9,12-octadecadienoate, and L-ascorbyl dipalmitate), palmitic acid, 4-ethyl-2-methoxyphenol, acetoin, and 2-ethyl-3,5-dimethylpyrazine.

## 4. Discussion

High-temperature *Daqu* is a crucial saccharification and fermentation starter for baijiu, and its quality determines the yield and quality of sauce-flavored baijiu [38]. One of the main factors contributing to the distinctive aroma of northern Chinese sauce-flavored baijiu is the high-temperature *Daqu,* which plays a key role in the manufacturing process. Therefore, the present study comprehensively analyzed the physicochemical indexes, volatile flavor components, and microbial community compositions of three kinds of high-temperature *Daqu*, which offers a more thorough and scientific assessment of *Daqu* for production applications, and also provides a scientific foundation for the processing standardization of northern Chinese sauce-flavored baijiu. 

The physicochemical indexes of *Daqu* determined its quality and whether it was suitable for fermentation. During the *Daqu* production process, the moisture content should not be more than 13.0% [17]. As displayed in Table 1, the moisture content of three *Daqu* samples met these standards. BQ showed the highest moisture, and WQ showed the lowest moisture because of the free- and well-ventilated storage conditions, and WQ was located in the outer layer, resulting in more moisture loss, while BQ was located in the inner layer, with less moisture loss [39]. However, this result was not completely consistent with the southern Chinese high-temperature *Daqu* [35]. The divergence of moisture in *Daqu* was mainly due to the differences in climate and humidity of the fermentation environments in the north and south of China [4]. The research showed that the acidity of high-temperature *Daqu* should be approximately 1.0 [40], and the acidities of the three *Daqu* types were in accordance with the acidity indexes. The saccharifying and liquefying powers refer to the ability of microorganisms with saccharifying functions in *Daqu* to convert starch into sugar [41]. The microorganism metabolizing the saccharifying function of hydrolase in *Daqu* is mainly *Aspergillus* [17]. Furthermore, the results of the microbial community composition analysis showed that *Bacillus* and *Aspergillus* were the most prevalent in the BQ sample (Figure 3), so it was indicated that BQ showed the highest saccharifying power and the lowest starch, while WQ had the highest liquefying power and starch. The results of previous studies showed a different phenomenon where RQ and YQ showed the highest saccharifying power in the different colored high-temperature *Daqu* of Sichuan, and WQ showed the highest saccharifying power in the different colored high-temperature *Daqu* of Guizhou [35,39,41]. The esterification power of *Daqu* represents the capacity of microbe-produced esterification enzymes to produce esters. [42]. The experimental results showed that the highest esterifying power of BQ was 288.36 U, and it was indicated that BQ has a strong ability to synthesize esters.

The volatile components in the three *Daqu* samples were detected using HS-SPME-GC-MS. A total of 96 volatiles were detected in the three samples; BQ contained 55, WQ contained 59, and YQ contained 61. The primary volatile flavor compounds in the *Daqu* samples were esters, alcohols, acids, phenols, ketones, and pyrazines (Table S1). Esters, often with fruity and flowery aromas, were thought to be the main contributors to the aromatic character of *Daqu* samples [43]. One of the four main ester components in baijiu is ethyl acetate, which is also the main ester in sauce-flavored baijiu and has a fruity flavor [4,44]. As displayed in Table 2, the content of ethyl acetate was the highest among the more than ten ester compounds identified in the three *Daqu* samples. YQ had the highest amount of ethyl acetate, but BQ had a higher concentration. It was indicated that ethyl acetate might be mainly produced by YQ in high-temperature *Daqu*. In addition, contents of hexadecanoic acid ethyl ester, n-propyl 9,12-octadecadienoate, and phenethyl acetate were high in the YQ sample. Hexadecanoic acid ethyl ester has a creamy and fruity aroma. Ethyl linoleate is the main ingredient of the empty cup flavor of sauce-flavored baijiu, and it has a good effect on hypertension, coronary heart disease, and other vascular diseases [45]. Fan et al. [42] reported this compound for the first time in baijiu. N-propyl 9,12-octadecadienoate was rarely identified in baijiu, so it is vital to investigate how it affects the flavor of baijiu. Phenethyl acetate contributes to the rosy aroma; it is an important aromatic compound in sauce-flavored baijiu [46]. L-ascorbyl dipalmitate was discovered in high-temperature *Daqu* for the first time and was not reported in other *Daqu* samples previously. Additional research on its contribution to baijiu flavor is necessary. Among the alcohols detected in the three *Daqu* samples, the contents of phenethyl alcohol and 2,3-butanediol were the highest, and phenethyl alcohol has rosy, fruity, and sweet bread aromas, and 2,3-butanediol has a sweet aroma [47]. Thus, it is speculated that these two substances were crucial aromatic compounds in high-temperature *Daqu*. Benzyl alcohol has jasmine oil and hyacinth aromas [46]. In addition, acetic acid (vinegar) and butanoic acid were the main aromatic compounds in sauce-flavored baijiu [43]; acetic acid and alcohol could be further synthesized into esters via an esterification reaction [48]. Acetoin is a precursor of tetramethylpyrazine, which has a strong creamy and fatty aroma, and they provide important flavors for baijiu [49]. Pyrazines have roasted peanut, hazelnut, cocoa, and nutty aromas, and they were detected in sauce-flavored baijiu in higher proportions than other flavored types of baijiu. [27]. Furthermore, tetramethylpyrazine has medical effects, such as treating cardiovascular diseases and improving learning disabilities [50,51]. In this study, the contents of pyrazines in three kinds of *Daqu* were the highest, and tetramethylpyrazine was the most abundant, comprising 31–46% of the total pyrazines. Additionally, the most abundant types of pyrazines were found in WQ (Figure 2). Nevertheless, the pyrazine content was significantly lower compared to the southern high-temperature *Daqu* [27,48]. 

The flavor compounds were formed via numerous metabolic reactions in *Daqu*, and they were largely affected by complex microbial communities. Furthermore, there are enormous differences in microbial community diversity among the three samples, which may be related to the *Daqu* production process, temperature, environment, and other factors. The microbial diversity analysis results showed that compared with the WQ sample, BQ and YQ showed higher microbial species richness and diversity (Table 2). The results of the microbial community composition analysis showed that the two most common bacterial phyla were Firmicutes and Actinobacteria, and the most common fungus phylum was Ascomycota (Figure 3a,c), which is consonant with previous reports [17]. They are not only the dominant microorganisms in high-temperature *Daqu* but also the dominant microorganisms in other baijiu *Daqu* [52]. *Bacillus*, *Kroppenstedtia*, *Virgibacillus*, *Oceanobacillus*, and *Scopulibacillus* were predominant bacterial genera, and *Thermoascus*, *Thermomyces*, *Byssochlamys*, and *Aspergillus* were dominant fungal genera in the three *Daqu* samples (Figure 3b,d). These results were different from previous studies in some southern regions, owing to the differences in climate conditions between the north and the south; the south is richer in the composition of brewing microorganisms [15,17,48]. It was demonstrated that the microorganism composition in high-temperature *Daqu* was distinctive between different producing regions and generated different microbial community structures. In this research, the composition of the fungal microbial community was markedly lower than the bacterial microbial community, which was mostly due to the thermolabile yeastand themold were eliminated when the fermentation temperature increased during the manufacturing process of high-temperature *Daqu* [16,17].

The *Bacillus* and *Aspergillus* genera were the most abundant in the BQ sample (Figure 3b,d). However, in the study of southern Chinese high-temperature *Daqu*, the predominant genus of WQ was *Bacillus* [48]. *Bacillus* and *Aspergillus* were the main microbial genera in various types of *Daqu*, which could produce a variety of flavor precursors and substances; especially important are contributions to the production of pyrazines [15,53,54]. Interestingly, the correlational heatmap analysis showed that *Bacillus* and *Aspergillus* had no significant correlation with tetramethylpyrazine. The result suggested that the driving force for the increase in the tetramethylpyrazine concentration in *Daqu* was not microorganisms but was possibly influenced instead by temperature and other environmental factors [55]. Previous studies have identified *Byssochlamys* as a dominant fungal genus in high-temperature *Daqu*, and *Byssochlamys* could degrade starch in raw materials [35]. The relative abundance of *Byssochlamys* was significantly higher in BQ than in WQ and YQ (Figure 3d), and the abundance of *Byssochramys_spectabilis* was also the highest (Figure S 4a), which may be owing to the higher fermentation temperature of BQ. In this study, the RDA analysis also showed that *Aspergillus* and *Byssochlamys* were significantly positively correlated with saccharifying and fermenting powers (Figure 5). *Oceanobacillus* promotes the production of esterase and amylase [25], which was the dominant bacteria genus in YQ and BQ, and relative abundance was higher in YQ than in BQ (Figure 3c). Wang et al. found that *Oceanobacillus* was a dominant bacteria in sauce-flavored baijiu fermentation [21], along with Luzhou-flavored baijiu and Fen-type fermented grains [52,56]. Shi et al. showed that *Oceanobacillus* was the dominant genus in the WQ type of southern high-temperature *Daqu*, while the abundance of *Oceanobacillus* in WQ in this study was less than 1% [27]. Figure 6 showed that *Oceanobacillus* and *Byssochlamys* were positively correlated with some esters, alcohols, acids, and tetramethylpyrazine. Many studies have identified *Thermoascus* and *Thermomyces* as dominant fungi in different *Daqu* samples [48,57]. *Thermoascus* could produce various enzymes and have a crucial impact on the formation of high-temperature *Daqu* [58]. However, the correlation between *Thermomyces* and flavor was completely opposite to that of *Byssochlamys*; it was positively correlated with pyrazines instead (Figure 6). *Byssochlamys*, *Thermoascus*, and *Thermomyces* belong to thermostable microorganisms, which have high abundance in YQ and BQ. *Scopulibacillus* and *Kroppenstedtia* had the highest relative abundance in WQ (Figure 3b). The RDA analysis showed that *Scopulibacillus* demonstrated a huge positive connection with starch (Figure 5), and starch was the highest in WQ; the results were consistent. However, the correlational analysis showed that *Kroppenstedtia* had no significant correlation with the physicochemical indexes and volatile flavor components (Figure 5 and Figure 6). Jiang et al. also demonstrated that *Scopulibacillus* and *Kroppenstedtia* were predominant genera in northern Jiang-favored *Daqu*. However, little research has been conducted on their biological functions [59]. *Kroppenstedtia* belongs to the *Thermoactinomyces* family, and many studies have found that it is a dominant bacteria in different *Daqu* samples [52]. In the late phase of fermentation of *Daqu*, *Kroppenstedtia*, *Bacillus*, and *Scopulibacillus* significantly increased [55]. *Virgibacillus* had the highest abundance in YQ. Zhang et al. [60] determined that *Virgibacillus* also was the dominant bacterial strain in Gujinggong baijiu *Daqu*. In the study, the correlational analysis of *Virgibacillus* and *Kroppenstedtia* showed similar results (Figure 5 and Figure 6). Nevertheless, *Virgibacillus* in yellow_Qu of southern high-temperature *Daqu* exhibited positive correlations with most volatile flavor components [27]. This result was contrary to this study, which may be one of the reasons affecting the different flavors of the southern and northern Chinese sauce-flavored baijiu. These results indicate that although these microorganisms were the prominent genera in high-temperature *Daqu*, they had a negative relationship with the main volatile flavor substances, suggesting that the microorganisms had no remarkable influence on the aroma formation, but they played a key role in balancing the interaction between microorganisms. Moreover, other genera, such as *Saccharopolyspora* and *Staphylococcus*, showed a significant positive correlation with most flavors, while *Monascus* showed a strong direct association with esterification power (Figure 5). *Saccharopolyspora* and *Staphylococcus* were the dominant genera in YQ, and *Monascus* was the dominant genus in BQ, but their abundance was far lower than that of southern Chinese high-temperature *Daqu* [15,16,27,35].

**Figure 7:**
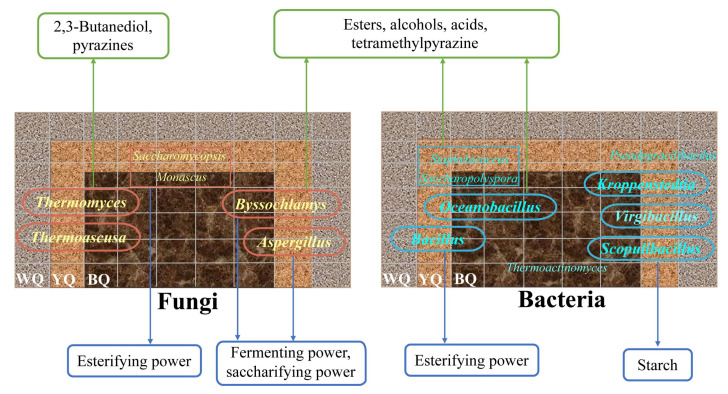
A profile map of the three colors of high-temperature *Daqu* with the dominant microbial and potential functions.

To sum up, a profile of three colors of high-temperature *Daqu* was drawn based on the obtained results from this work (Figure 7) in order to visualize the connection between the dominant microbiological and physicochemical indexes and volatile components in three types of *Daqu*. As we all know, in the actual production of sauce-flavored baijiu, the use of the three colors of high-temperature *Daqu* is the distillery master with years of operating experience in accordance with the different color ratios before baijiu brewing; they are not clear about the function of the three colors of high-temperature *Daqu*. Our study offers a crucial scientific foundation for the actual production of three colors of high-temperature *Daqu* in the northern region of Chinese sauce-flavored baijiu. The above studies explained a portion of the variations in the microbial characteristics and potential functions in three different colors of high-temperature *Daqu*; metabolomics technologies should be performed to further clarify the role of these microorganisms in the fermentation process of *Daqu* in order to explore the differences between the high-temperature *Daqu* of northern and southern China.

## 5. Conclusions

In this work, we systematically and comparatively analyzed the physicochemical indexes, volatile flavor compounds, and microbial community compositions of the three different colors of high-temperature *Daqu* in northern China. WQ exhibited the highest liquefying power and starch, while BQ showed the highest saccharifying and esterifying powers. The microbial community structure analysis showed marked differences in the structures and potential functions of bacterial and fungal communities among the different colors of *Daqu*. *Bacillus*, *Kroppenstedtia*, *Virgibacillus*, *Oceanobacillus,* and *Scopulibacillus* were predominant bacterial genera, and *Thermoascus*, *Thermomyces*, *Byssochlamys*, and *Aspergillus* were dominant fungi genera. The RDA analysis revealed the correlation between the dominant microorganisms and different physicochemical indexes. The Spearman correlational analysis indicated that *Oceanobacillus*, *Saccharopolyspora*, *Staphylococcus*, *Pseudogracilibacillus*, *Byssochlamys*, and *Thermomyces* showed positive correlations with part of the majority of key volatile flavor compounds. However, *Thermomyces* was negatively correlated with most compounds. This work clearly illustrates the compositions and functions of three colors of high-temperature *Daqu* in northern China; the results obtained provide a scientific basis for the production standardization of sauce-flavored baijiu in northern China.

## Figures and Tables

**Table 1:** Physicochemical index analysis on three *Daqu* samples.

Sample	Moisture (%)	Acidity (mmol/10 g)	Starch (%)	Saccharifying Power (U)	Liquefying Power (U)	Esterifying Power (U)	Fermenting Power (U)
BQ	13.28 ± 0.18 ^a^	1.26 ± 0.07 ^a^	59.26 ± 0.33 ^c^	180.80 ± 7.16 ^a^	0.13 ± 0.04 ^b^	288.36 ± 14.61 ^a^	0.30 ± 0.02 ^a^
WQ	10.99 ± 0.07 ^c^	1.264 ± 0.05 ^a^	64.71 ± 0.69 ^a^	113.10 ± 2.50 ^c^	0.18 ± 0.01 ^a^	223.52 ± 14.90 ^b^	0.23 ± 0.01 ^a^
YQ	12.19 ± 0.16 ^b^	1.319 ± 0.17 ^a^	62.16 ± 0.50 ^b^	144.40 ± 2.71 ^b^	0.12 ± 0.02 ^b^	198.37 ± 17.50 ^c^	0.27 ± 0.01 ^a^

The different letters indicate significant differences in the physicochemical indexes between the three samples (*p* < 0.05).

**Table 2:** Species α-diversity indexes among three *Daqu* samples.

Species	Sample	Richness Indexes			Diversity Indexes		
		Chao1	ACE	Coverage	Shannon	Simpson	Coverage
Bacteria	BQ	291.55 ± 35.07 ^a^	306.37 ± 21.89 ^a^	0.999	2.08 ± 0.34 ^a^	0.36 ± 0.04 ^a^	0.999
	WQ	122.70 ± 10.37 ^b^	141.17 ± 15.94 ^b^	0.999	1.38 ± 0.02 ^a^	0.43 ± 0.03 ^a^	0.999
	YQ	352.39 ± 12.55 ^a^	361.60 ± 19.44 ^a^	0.999	2.67 ± 0.14 ^a^	0.19 ± 0.02 ^b^	0.999
Fungi	BQ	59.83 ± 2.95 ^a^	60.80 ± 3.00 ^a^	0.999	1.52 ± 0.01 ^a^	0.32 ± 0.01 ^a^	0.999
	WQ	48.38 ± 0.82 ^b^	57.81 ± 0.68 ^a^	0.999	1.29 ± 0.15 ^a^	0.36 ± 0.01 ^a^	0.999
	YQ	60.83 ± 1.06 ^a^	69.12 ± 3.42 ^a^	0.999	1.04 ± 0.07 ^a^	0.43 ± 0.05 ^a^	0.999

The different letters indicate significant differences in the physicochemical indexes between the samples (*p* < 0.05).

## Data Availability

The data are contained within the article or the Supplementary Materials.

## Supplementary Materials

The following supporting information can be downloaded at: https://www.mdpi.com/article/10.3390/foods12020326/s1, Figure S1. Microbial community composition of each sample. (a). Diagram for bacteria in species level (b) Diagram for fungi in species level; Table S1. Volatile flavor components identified by HS-SPME-GC-MS in three colors of high-temperature *Daqu*.
