# Supplementary material for: Correlational Analysis of the Physicochemical Indexes, Volatile Flavor Components, and Microbial Communities of High-Temperature Daqu in the Northern Region of China
Source: Foods. 2023 Jan 9;12(2):326. doi: 10.3390/foods12020326 (PMC9857448; doi:10.3390/foods12020326)
Supplement: Supplementary file 1 [file foods-12-00326-s001.zip › foods-2052328-supplementary.docx]

**Supplementary material**

**Correlation Analysis of Physicochemical Indexes, Volatile Fla-vor Components and Microbial Communities of High-Temperature Daqu in North Region of China**

Zemin Pang 1ǂ, Weiwei Li 2ǂ, Jing Hao 1, Youqiang Xu 2, Binghao Du 2, Chengnan Zhang 2, Kun Wang 3, Hua Zhu 3, Hongan Wang 3, Xiuting Li 2*, Changhong Guo 1*

1 Key Laboratory of Molecular and Cytogenetic of Heilongjiang Province & College of Life Science and Technology, Harbin Normal University, Harbin 150025, China; pzmin920@163.com (Z.P.); 1783848100@qq. com (J.H.)

2 Key Laboratory of Brewing Microbiome and Enzymatic Molecular Engineering, China General Chamber of Commerce, Beijing Technology and Business University, Beijing 100048, China. liweiwei.0304@163.com (W.L.); xuyouqiang@btbu.edu.cn (Y.X.); BinghaoDu@163.com (B.D.); zhangcn@btbu.edu.cn (C.Z.)

3 Beijing Huadu Brewery & Food Industry Co., Ltd, Beijing 102212, China. wk@huadujiuye.cn (K.W.); 51044936@qq.com (H.Z.); 1460369765@qq.com (H.W.)

* Correspondence: kaku3008@hrbnu.edu.cn (C.G.); lixt@btbu.edu.cn (X.L.)

ǂThese authors contributed equally to this work.

Supplementary Table

Table S1. Volatile flavor components identified by HS-SPME-GC-MS in three colors of high-temperature *Daqu*.

| **Compounds ID** | **Compounds** | **Concentration (μg/kg)** | | |
| --- | --- | --- | --- | --- |
|  |  | **BQ** | **WQ** | **YQ** |
|  | Total Compounds | 220.16±33.75 | 203.27±40.52 | 298.93±48.93 |
|  | Esters (19) | 23.74±3.11 | 16.11±2.15 | 71.86±12.11 |
| C1 | Ethyl acetate | 8.76±2.44 | 1.14±0.02 | 10.65±3.32 |
| C2 | Ethyl caprylate | nd | nd | 3.71±0.72 |
| C3 | Phenethyl acetate | 2.39±0.11 | 0.08±0.01 | 12.77±2.24 |
| C4 | Ethyl myristate | 1.63±0.07 | 0.81±0.03 | 2.25±0.12 |
| C5 | Hexadecanoic acid ethyl ester | 3.92±0.25 | 5.08±1.73 | 19.78±4.53 |
| C6 | Ethyl linoleate | 1.16±0.02 | 0.75±0.02 | 0.71±0.02 |
| C7 | Ethyl 9-octadecenoate | 1.04±0.01 | 1.85±0.05 | 6.19±0.11 |
| C8 | Ethyl cinnamate | nd | nd | 0.19±0.02 |
| C9 | n-Propyl 9,12-octadecadienoate | 1.29±0.10 | 2.91±0.13 | 13.03±0.71 |
| C10 | Ethyl 9-hexadecenoate | 2.12±0.02 | 0.71±0.01 | 2.17±0.11 |
| C11 | Ethyl 9-cis-11-trans-octadecadienoate | 0.28±0.02 | 0.20±0.01 | 0.65±0.08 |
| C12 | Ethyl 9,12,15-octadecatrienoate | nd | 0.26±0.02 | 0.87±0.05 |
| C13 | Ethyl 13-methyl-tetradecanoate | 0.26±0.03 | 0.67±0.03 | nd |
| C14 | Tetradecanoic acid-14-oxo-ethyl ester | nd | 0.13±0.01 | nd |
| C15 | γ-Nonanolactone | 0.47±0.01 | nd | nd |
| C16 | L-ascorbyl dipalmitate | 0.42±0.03 | 0.92±0.04 | 1.18±0.05 |
| C17 | 2-Octylcyclopropanetetradecanoic acid methyl ester | nd | nd | 0.16±0.02 |
| C18 | 2'-Hexyl-1,1'-bicyclopropane-2-octanoic acid methyl ester | nd | 0.11±0.01 | 0.09±0.01 |
| C19 | Methyl anthranilate | nd | 0.49 ±0.03 | nd |
|  | Alcohols (16) | 65.25±8.23 | 55.49±9.40 | 90.66±13.73 |
| C20 | 1-Pentanol | 7.44±0.21 | 7.65±0.77 | 9.54±3.04 |
| C21 | Isoamyl alcohol | nd | 8.90±0.50 | 0.75±0.02 |
| C22 | n-Hexanol | 7.11±0.20 | 2.33±0.02 | 4.38±0.01 |
| C23 | 2,3-Butanediol | 13.02±3.36 | 17.13±4.18 | 7.63±1.55 |
| C24 | Benzylalcohol | 2.41±0.16 | nd | 3.92±0.40 |
| C25 | Phenethyl alcohol | 32.33±4.24 | 17.94±3.83 | 57.64±8.31 |
| C26 | 2-Methyl-1-hexadecanol | 0.91±0.01 | nd | 0.80±0.01 |
| C27 | 1-Undecanol | 1.14±0.02 | nd | nd |
| C28 | 2-Undecanol | nd | nd | 2.20±0.21 |
| C29 | Undecaethylene glycol | 0.66±0.02 | 0.81±0.03 | 0.60±0.04 |
| C30 | 3-Methyl-2-hexanol | nd | nd | 1.31±0.05 |
| C31 | 2-Octanol-3-methyl | nd | nd | 1.61±0.07 |
| C32 | Linalool | nd | 0.21±0.02 | nd |
| C33 | á-Ethylphenethyl alcohol | 0.23±0.01 | 0.33±0.04 | nd |
| C34 | 1-Hexadecanol | nd | nd | 0.28±0.02 |
| C35 | à-Methyl-benzeneethanol | nd | 0.19±0.01 | nd |
|  | Acids (16) | 14.88±1.53 | 12.12±0.71 | 18.67±1.52 |
| C36 | Acetic acid | 4.98±1.01 | 3.26±0.05 | 9.20±1.23 |
| C37 | Hexanoic acid | 1.04±0.06 | nd | 0.60±0.02 |
| C38 | Butyric acid | 2.68±0.10 | nd | nd |
| C39 | Isobutyric acid | nd | 0.36±0.03 | nd |
| C40 | 3-Methyl-butanoic acid | 2.27±0.05 | nd | 2.73±0.03 |
| C41 | 2-Methyl butyric acid | nd | 4.58±0.06 | nd |
| C42 | 4-Methylvaleric acid | nd | 1.57±0.08 | nd |
| C43 | 3-Methylvaleric acid | 0.84±0.03 | 0.47±0.01 | 2.35±0.12 |
| C44 | Palmitic acid | 0.66±0.01 | 0.36±0.02 | 1.24±0.03 |
| C45 | 4-Hydroxybenzenephosphonic acid | 0.68±0.23 | 1.12±0.42 | 1.55±0.01 |
| C46 | Gamolenic acid | nd | 0.11±0.01 | 0.16±0.01 |
| C47 | Arachidonic acid | nd | 0.14±0.01 | 0.23±0.02 |
| C48 | 3-Hydroxydodecanoic acid | 0.62±0.01 | nd | nd |
| C49 | 3-Methyloxirane-2-carboxylic acid | 1.11±0.03 | nd | nd |
| C50 | Doconexent | nd | 0.15±0.02 | nd |
| C51 | 2,3-Dimethyl-2-pentenoic acid | nd | nd | 0.61±0.05 |
|  | Aldehydes (5) | 2.34±0.09 | 3.58±0.43 | 2.31±0.14 |
| C52 | Benzaldehyde | 1.45±0.06 | 3.58±0.43 | 1.32±0.08 |
| C53 | 3-(Furan-2-yl)-2-phenylprop-2-enal | 0.18±0.01 | nd | 0.11±0.02 |
| C54 | 1H-Pyrrole-2-carboxaldehyde | 0.71±0.02 | nd | 0.41±0.02 |
| C55 | 4-Methyl-hexanal | nd | nd | 0.25±0.01 |
| C56 | 2-Phenyl butyric anhydride | nd | nd | 0.22±0.01 |
|  | Phenols (7) | 7.67±0.18 | 6.28±1.45 | 9.95±1.52 |
| C57 | Phenol | 1.82±0.02 | nd | 2.07±0.13 |
| C58 | 4-Ethylphenol | 2.12±0.01 | 0.26±0.01 | nd |
| C59 | 4-Ethyl-2-methoxyphenol | nd | 1.49±0.02 | 4.03±1.16 |
| C60 | 2-Methoxy-4-vinylphenol | 1.79±0.11 | 3.50±1.41 | 3.03±0.21 |
| C61 | 2-Naphthalenol | 0.78±0.01 | 1.03±0.01 | nd |
| C62 | Creosol | 0.61 ±0.02 | nd | nd |
| C63 | 2,4-Di-tert-butylphenol | 0.55 ±0.01 | nd | 0.82 ±0.02 |
|  | Ketones (5) | 5.74±0.61 | 8.52±1.44 | 6.12±3.62 |
| C64 | Acetoin | nd | 4.72±1.02 | 6.12±3.62 |
| C65 | 1-Acetoxy-p-menth-3-one | 1.22±0.07 | nd | nd |
| C66 | 4-Octanone | 4.52±0.54 | 2.88±0.36 | nd |
| C67 | 1-(4-Methoxy-phenyl)-3-methyl-pentan-1-one | nd | 0.21±0.03 | nd |
| C68 | (Z)-Oxacyclopentadec-6-en-2-one | nd | 0.71±0.03 | nd |
|  | Pyrazines (7) | 93.56±19.77 | 94.12±24.41 | 90.15±15.89 |
| C69 | 2,5-Dimethylpyrazine | 25.22±5.11 | 11.20±3.64 | 21.19±4.23 |
| C70 | 2,6-Dimethylpyrazine | 23.11±4.12 | nd | nd |
| C71 | 2-Ethyl-3,5-dimethylpyrazine | nd | 12.00±4.05 | 12.45±1.91 |
| C72 | 2-Butyl-3,5-dimethylpyrazine | nd | 10.80±3.04 | nd |
| C73 | Tetramethylpyrazine | 45.23±10.54 | 38.41±9.14 | 56.51±9.75 |
| C74 | 2,5-Dimethyl-3-(3-methylbutyl)-pyrazine | nd | 11.11±3.02 | nd |
| C75 | 2,5-Dimethyl-3-n-pentylpyrazine | nd | 10.6±2.02 | nd |
|  | Ethers (4) | 4.41±0.09 | 2.98±0.11 | 1.94±0.16 |
| C76 | Octaethylene glycol monododecyl ether | 0.26±0.02 | 0.06±0.01 | 0.14±0.02 |
| C77 | Heptaethylene glycol monododecyl ether | 0.16±0.04 | 0.17±0.08 | 0.32±0.07 |
| C78 | Dimethyl trisulfide | 2.70±0.01 | 1.20±0.01 | 0.40±0.03 |
| C79 | 1,2-Dimethoxybenzene | 1.29±0.02 | 1.55±0.01 | 1.08±0.04 |
|  | Others (17) | 2.57±0.14 | 4.07±0.42 | 3.73±0.24 |
| C80 | 1-(1H-pyrrol-2-yl)-Ethanone | 0.41±0.04 | nd | 0.25±0.02 |
| C81 | 3-Acetyl-1H-pyrroline | 0.63±0.01 | nd | 0.27±0.01 |
| C82 | 2,3-Dihydrobenzofuran | 0.37±0.03 | 0.82±0.35 | 1.18±0.05 |
| C83 | 3-Phenyl-Furan | nd | 0.11±0.02 | nd |
| C84 | 4,6-Dimethylpyrimidine | 0.32±0.01 | nd | 0.36±0.06 |
| C85 | 2-Phenylpyridine | nd | nd | 0.31±0.02 |
| C86 | 6-Phenylundecane | 0.23±0.01 | nd | nd |
| C87 | Nitrosomethane | nd | 0.21±0.01 | nd |
| C88 | 3,4-Dimethyl-Heptane | nd | nd | 0.61±0.04 |
| C89 | (2-Methylbutyl) oxirane | nd | nd | 0.13±0.01 |
| C90 | 2-Methylstyrene | 0.25±0.01 | nd | nd |
| C91 | 2-methyl-1-Octene | nd | 0.23±0.01 | nd |
| C92 | 1,2,3-Trimethoxybenzene | nd | 1.05±0.02 | nd |
| C93 | 1,2,4-Trimethoxybenzene | nd | 1.65±0.01 | nd |
| C94 | (2-Aziridinylethyl) amine | nd | nd | 0.48±0.02 |
| C95 | 2-methyl-4-propyl-Oxetane | nd | nd | 0.14±0.01 |
| C96 | Indolizine | 0.36±0.03 | nd | nd |

nd, not detected.

Supplementary Figure


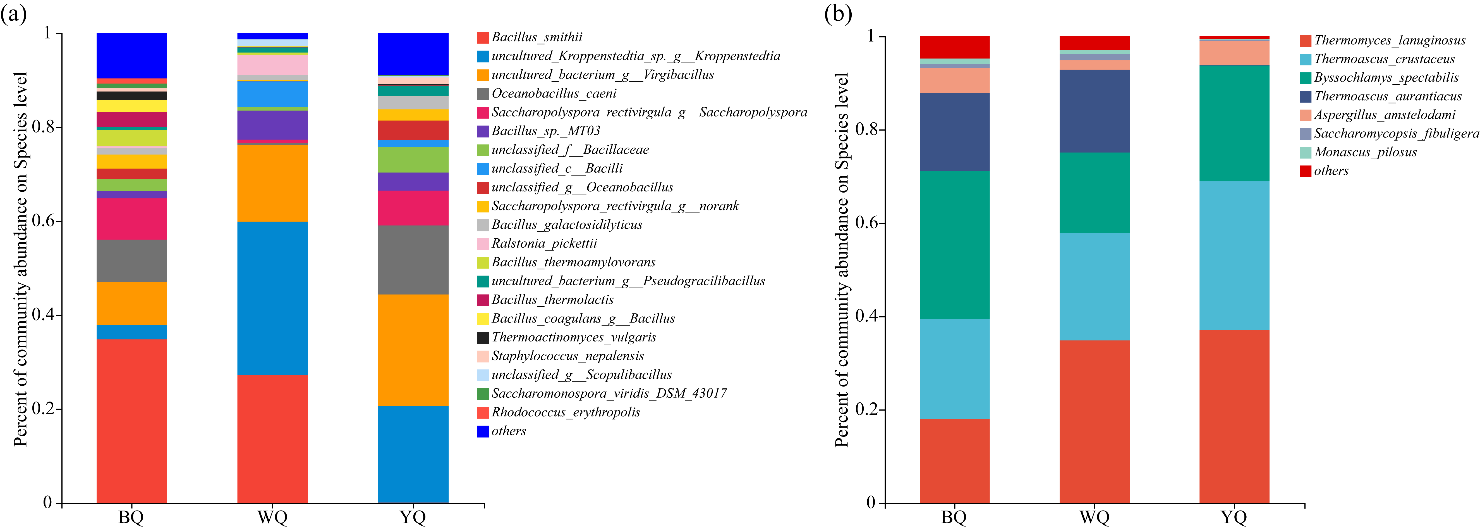


Figure S1. Microbial community composition of each sample. (a). Diagram for bacteria in species level (b) Diagram for fungi in species level.
